# Humans, but not their dogs, displace pumas from their kills: An experimental approach

**DOI:** 10.1038/s41598-019-48742-9

**Published:** 2019-08-21

**Authors:** Justin P. Suraci, Justine A. Smith, Michael Clinchy, Liana Y. Zanette, Christopher C. Wilmers

**Affiliations:** 1 Center for Integrated Spatial Research, Environmental Studies Department, University of California, Santa Cruz, CA 95064 USA; 2 Department of Environmental Science, Policy, and Management, University of California, Berkeley, CA 94720 USA; 3 Department of Biology, Western University, London, ON N6A 5B7 Canada

**Keywords:** Behavioural ecology, Conservation biology

## Abstract

Domestic dogs are the most abundant large carnivore on the planet, and their ubiquity has led to concern regarding the impacts of dogs as predators of and competitors with native wildlife. If native large carnivores perceive dogs as threatening, impacts could extend to the community level by altering interactions between large carnivores and their prey. Dog impacts may be further exacerbated if these human-associated predators are also perceived as indicators of risk from humans. However, observational approaches used to date have led to ambiguity regarding the effects of dog presence on wildlife. We experimentally quantified dog impacts on the behavior of a native large carnivore, presenting playbacks of dog vocalizations to pumas in central California. We show that the perceived presence of dogs has minimal impacts on puma behavior at their kill sites, and is no more likely to affect total feeding time at kills than non-threatening controls. We previously demonstrated that pumas exhibit strong responses to human cues, and here show that perceived risk from human presence far exceeds that from dogs. Our results suggest that protected areas management policies that restrict dogs but permit human access may in some cases be of limited value for large carnivores.

## Introduction

Through their association with humans, domestic dogs (*Canis lupus familiaris*) have become the most abundant large carnivore on the planet^1^, and their ubiquity has led to concern regarding the impacts of dogs as predators of and competitors with native wildlife^1–4^. Direct predation by dogs is a potential threat to some wildlife populations, particularly where large numbers of feral or free-roaming dogs occur^3,5–8^. However, non-consumptive effects of dogs may be even more pervasive if perceived risk causes native wildlife to be displaced from valuable habitat and resources, or interferes with foraging and reproductive behavior^8–10^. Such non-consumptive effects may extend to impact entire communities, particularly if dog presence affects native large carnivores, which in some systems play a crucial role in structuring ecosystems through their top-down effects on smaller predators and herbivores^11–13^. Perceived risk from heterospecific competitors has been shown to alter large carnivore feeding and habitat use^14–17^, meaning that, if native large carnivores perceive domestic dogs as threatening competitors, dog-induced behavioral changes could cascade across terrestrial communities by interfering with the native predator’s ability to regulate its prey. However, ambiguity exists as to whether dogs pose a substantial threat to most native large carnivores. Indeed, rather than competitors, dogs may be prey to some large carnivore species^1,18^.

In addition to their potential impacts as predators and competitors, the strong association between dogs and humans may mean that dog cues (e.g., the vocalizations of this highly vocal species) are perceived by wildlife as a reliable indicator of risk from humans. A similar phenomenon has been demonstrated in moose, which respond to ravens where ravens are a reliable indicator of the presence of wolves^19,20^. Perception of dogs as a proxy for humans could lead to particularly strong impacts of dog presence on wildlife populations subject to human hunting. This may be especially pertinent when considering impacts on native large carnivores, species for which humans are often a primary source of mortality^21–23^. Fear of humans has been shown to elicit strong behavioral responses in native large carnivores^24–27^, which, if generalized to dogs, could increase the impacts of dog presence on native carnivore behavior.

Much of our knowledge regarding the non-consumptive effects of domestic dogs on large carnivores and other wildlife comes from studies in protected areas (reviewed in^28^), where dog impacts have been a topic of considerable management concern^29^. However, these studies provide conflicting results, variously reporting substantial displacement of wildlife where dogs are present^2,30,31^, or little to no effect of dogs on wildlife^32,33^. This uncertainty regarding dog impacts extends to native large carnivores, including pumas (*Puma concolor*), which studies have suggested either avoid^4,31^ or are indifferent to^32,33^ the presence of dogs. Most studies to date (including all large carnivore studies just mentioned) have taken an observational approach, comparing camera trap- or scat-derived estimates of wildlife habitat use between protected areas where dogs are permitted with their owners and those where dogs are restricted. Conflicting conclusions regarding the impacts of dogs may therefore stem from limited capacity to control for alternative influences on wildlife behavior that differ between protected areas. Furthermore, such study designs are unable to differentiate between impacts of dogs as predators/competitors versus dogs as indicators of risk from humans. These issues highlight the need for experimental approaches to tease apart the potentially complex effects of domestic dogs on large carnivores and other native wildlife^34,35^.

Here we use the experimental presentation of dog vocalizations to quantify the non-consumptive effects of dogs on a native large carnivore, the puma. Pumas in the Santa Cruz Mountains of central California inhabit a matrix of wildlands and exurban and suburban development that is heavily used by both humans and domestic dogs^36,37^. Humans are a primary source of puma mortality in this region (CCW, unpublished data), and we previously demonstrated experimentally that pumas here exhibit strong behavioral responses to the perceived presence of humans^26,38^. Pumas were more likely to be displaced from their kills and to reduce overall feeding time in response to playbacks of human vocalizations, relative to non-threatening controls^26^. Humans therefore represent a known source of fear for pumas in our study population, allowing us to rigorously quantify the impacts of dogs in the present study by comparing puma responses to dogs with responses to humans (a known threat) and non-threatening controls. As described below, we also take advantage of differences in the level of potential threat posed by different dog breed sizes to further separate puma responses to dogs as competitors from responses to dogs as an indicator of human presence.

## Methods

Building on the findings of Smith *et al*.^26^, and following the experimental protocol described therein, we conducted a playback experiment with pumas at their active kill sites. While wildlife species respond to a variety of cue types, presentation of vocalizations provides the most reliable method of simulating the immediate presence of a predator or competitor^39^, and indeed dozens of studies have shown that wildlife exhibit strong behavioral responses to just the sounds of their enemies^40,41^. We tracked adult pumas fitted with GPS collars (GPS Plus and Vertex, Vectronics Aerospace, Berlin, Germany) sampling at 4-hour intervals, and identified potential fresh kill sites as clusters of two or more GPS locations occurring between sunset and sunrise and within 100 m of each other. We investigated potential kills sites, and if a fresh deer kill was found with sufficient meat remaining that a puma was likely to return on subsequent nights, we deployed camera and playback equipment in the immediate vicinity of the kill (as described below) and staked the deer carcass in place to prevent the puma from dragging it out of the camera’s field of view.

Smith *et al*.^26^ compared puma responses to human vocalizations with responses to a non-threatening control, Pacific tree frogs (*Pseudacris regilla*). Here, we use an identical methodology, presenting pumas with vocalizations of both domestic dogs and tree frogs. As described in Smith *et al*.^26^, tree frog vocalizations provide an ideal control because, like dogs and humans, tree frogs are common throughout our study area and can be heard both day and night, but unlike dogs and humans, frogs should represent no risk to pumas. As noted in the introduction, behavioral responses to the sound of dogs may be related either to (i) the threat posed by dogs themselves, or to (ii) the association between dogs and humans (i.e., dogs as a proxy for human threat). To distinguish between these possibilities, we compared puma responses to two classes of dogs: small (<7 kg) and large (>20 kg). Under the assumption that all sizes of dog are equally likely to be associated with humans, but only large dogs potentially pose a direct threat to pumas, detecting behavioral responses only to the playbacks of large dogs would indicate that the threat of dogs as competitors affects puma behavior, while detecting equally strong responses to both small and large dog playbacks would suggest that dogs are perceived as risky because of their association with humans. Following well established protocols^26,40,42^, we used multiple exemplars of each playback type (seven frog, six small dog, and five large dog), which were standardized to be broadcast at a consistent volume of 80 dB at 1 m (measured using Radioshack 33–2055 Digital Sound Level Meter set to fast response and C weighting). All dog playbacks consisted of a single individual barking. We composed 30 min playlists of each playback treatment by looping all exemplars of a given treatment.

**Figure 1:**
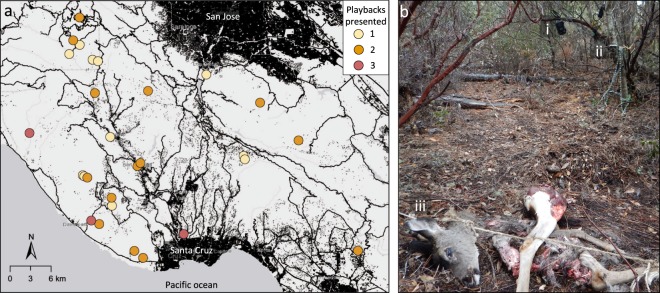
Puma playback sites. (**a**) Map of the spatial distribution of playback sites across the Santa Cruz Mountains study area. Locations of playback trials from both the present study and Smith *et al*.^26^ are included. Colors represent the number of different playback treatments presented at a given location (one to three). Black points and lines represent buildings and major roads, respectively. (**b**) Details of the experimental set-up at a single playback site, illustrating the integrated (i) speaker and (ii) camera trap of the Automated Behavioral Response system, and (iii) the fresh deer kill, staked in place to prevent the puma dragging the kill out of the cameras field of view.

We presented playbacks to pumas at their kill sites and video recorded their responses using the Automated Behavioural Response (ABR) system^43^ (Fig. 1). This motion sensitive playback system is triggered by animal movement, broadcasting a playback from a custom-built speaker and recording the animal’s response via an integrated camera trap. At each kill site, ABR speakers (modified EcoExtreme waterproof speaker, Grace Digital Inc., USA) were position 400–450 cm from the carcass and 150–200 cm above the ground. Integrated camera traps (Moultrie M990-I, Moultrie Products, LLC, USA) were position 300–350 cm from the carcass at a height of 70–130 cm. ABRs were programmed such that, when triggered, cameras recorded a 30 s video and speakers broadcast a 10 s playback during the middle 10 s of the video. Cameras were set to record sound, allowing us to determine which playback was broadcast during each trial. A puma that remained at the kill site could be exposed to the playback as frequently as twice per minute if continuous movement repeatedly triggered the ABR system. At each kill site, a second camera trap (Bushnell Trophy Cam, Bushnell Outdoor Products, USA), not integrated into the ABR system, was positioned approximately 180° from the primary camera to capture any behavioral responses by pumas that may have been missed by the primary camera. ABR mp3 players were programmed to continuously cycle through 30-min playlists of each playback treatment (i.e., 30-min of frogs, 30-min of small dogs, and 30-min of large dogs), and thus the treatment to which an individual puma was first exposed was random, being determined by when it first triggered the ABR. Depending on its response to the playbacks and total time spent at the carcass, an individual puma could be exposed to one, two, or all three playbacks at a given kill site (Fig. 1). Dog and frog playbacks were presented to 13 individual pumas (11 adults and 2 juveniles) between 10 October 2016 and 18 October 2017. All animal monitoring and experimental methods were approved by the University of California Animal Care and Use Committee (IACUC protocol WILMC_1612) and were conducted in accordance with guidelines and regulations of the State of California.

We compared puma responses to playbacks across three behavioral metrics: the probability of fleeing the kill site following the initial exposure to a given playback treatment, latency to return to the kill following initial exposure, and total time spent feeding during a given playback treatment over a 24-hr period. We compared puma responses to playbacks of large and small dogs with responses to (i) frogs and (ii) humans, using data on responses to human playbacks from Smith *et al*.^26^. These comparisons allowed us to test (i) whether pumas exhibit behavioral responses to perceived risk from large and/or small dogs, relative to non-threatening controls, and (ii) whether the magnitude of behavioral responses to large and/or small dogs is comparable to previously documented responses to humans, a known source of fear. As reported in Smith *et al*.^26^, all human playbacks (seven exemplars) consisted of a single individual speaking in a conversational tone. We performed all of the analyses involving frog playbacks using all available data, i.e., frog playback trials from both the present experiment and Smith *et al*. 2017[26] (n = 22 frog trials in total). Using pooled data provides increased statistical power to detect differences in responsiveness to frogs and dogs. Two individual pumas were exposed to frogs in both studies (i.e., at two separate kill sites), and only data from the first kill site are used here. Smith *et al*.^26^ report an average total time spent feeding during frog playbacks of 10.4 (±3.1 SE, n = 17) min, which was not significantly different from the value found in the present study (11.4 ± 4.1 min, n = 7; Mann-Whitney U-Test, *p* = 0.799), validating the use of pooled data from both studies. We also performed all analyses described below using only data from frog trials conducted during the present study and obtained similar results. Note that, because data on puma responses to human playbacks were taken from a previous experiment^26^ (conducted between 04 December 2015 and 19 June 2016), human playback trials necessarily preceded dog playback trials. However, only a subset of pumas was exposed to playbacks in both experiments. Across the two experiments, trials were conducted on 25 unique individuals, only five of which were exposed to cues in both experiments, with an average of 275 days (range 272–482 days) separating exposures from the two experiments. When considering treatment order across the two studies, 11 of the 25 unique individuals were exposed to potentially threatening sounds as their first playback (human = 4, large dog = 3, small dog = 4), while the remaining 14 were first exposed to frog controls. It is also worth noting that, because all kill sites were necessarily visited by humans to set up experimental equipment, cues of human disturbance may have been detectable regardless of playback treatment. Frog trials therefore provide a procedural control not only for the presentation of vocalizations, but also for any baseline disturbance detected by pumas at their kill sites.

All initial exposures to a given playback treatment were scored for whether or not the puma fled the kill site, defined as running away immediately following the playback. For every trial, we then scored the latency to “return” to the kill site following initial exposure to a playback treatment as the time in minutes between the video documenting the initial exposure and the subsequent video. We ranked all return time values, assigning the highest rank to trails in which the puma did not return. In some cases, pumas did not flee the kill site, but instead moved off slowly following a period of vigilance. In such cases it was possible for the trial to be scored as “did not return” if the puma never retriggered the camera following initial exposure. Finally, we quantified the amount of time that a puma was observed feeding from transcripts of all video footage, and calculated total time spent feeding while exposed to each playback treatment over the 24-hr period following initial exposure to that treatment.

For each playback trial, we collected data on additional, site-specific factors that could affect puma behavior at their kill sites using the spatial location of each kill (Fig. 1). We estimated (i) building density at each playback location as the number of buildings within 500 m and (ii) the straight-line distance from each playback location to the nearest major road. These spatial covariates were included in analyses of puma responses to playbacks (see below), and we also tested for systematic differences between treatments by modeling each spatial covariate as function of playback treatment using linear mixed effects models (LMM) with individual puma ID as a random effect. In these analyses, building density and distance to road were log transformed to meet normality assumptions. Tukey’s post-hoc tests were used to compare building density and distance to road between all pairs of playback treatments (Supplementary Table S1).

The effects of playback treatment on probability of fleeing the kill site and total time spent feeding over a 24-h period were analyzed using (generalized) LMM with either a binomial (fleeing) or normal (feeding time) error distribution, and including puma ID as a random effect, as several individual pumas were exposed to multiple playback treatments. Values for total time spent feeding were Box-Cox transformed to satisfy normality and homogeneity of variance assumptions^44^. For each response variable (fleeing or feeding), we first fit full models testing the effects of treatment (four levels: small dog, large dog, frog, human), building density, distance to road, and exposure (i.e., whether a given trial represented the first, second, etc. playback treatment to which the individual was exposed). This latter term was included to test whether the number of different playbacks to which a puma was exposed affected its probability of fleeing or total feeding time. We confirmed adequate model fit through visual inspection of residual vs. fitted value plots and quantile-quantile plots. We used likelihood ratio tests via Wald’s chi-squared statistic to find the best nested model (Supplementary Tables S2 and S3). Analysis of full models for both fleeing and feeding time indicated that only playback treatment significantly affected puma responses (see Results). We therefore fit reduced models and used Dunnett contrasts^45^ to simultaneously compare puma responses to large dogs and small dogs with responses to the treatment of interest (i.e., frogs or humans) while avoiding unnecessary comparisons (e.g., the human vs. frog comparison, which was previously presented by Smith *et al*.^26^). Full results of (G)LMM models and Dunnett contrasts are presented in Supplementary Tables S2 and S3. (G)LMMs were fit using the lme4 package in R^46^ and post-hoc tests and contrasts were analyzed using the multcomp package^47^.

Ranked values of latency to return to the kill were compared between treatments using Mann-Whitney U-tests. Because responses to both large and small dogs were compared to responses to frog and human playbacks, it was necessary to account for multiple comparisons using Bonferroni’s correction, and all p-values for Mann-Whitney U-tests were therefore multiplied by a factor of two. Because the U-test does not account for multiple observations from a single individual, we restricted our analyses of ranked latency values to only the first exposure of an individual puma to any playback treatment. Importantly however, neither restricting our data set in this way nor correcting for multiple comparisons affected the significance of our results regarding latency to return.

## Results

We obtained behavioral responses to large dog and small dog playbacks from 11 and 12 individual pumas, respectively. The number of individuals exposed to large and small dog playbacks was therefore comparable to the number exposed to human playbacks (n = 12) by Smith *et al*.^26^. Twenty-two individuals were exposed to frogs across the two studies. By chance, small dog trials were located slightly closer on average to major roads and buildings than were either frog or human trials (Supplementary Table S1), which could in principal have increased puma responsiveness during small dog trials due to additional disturbance. However, there is no evidence that this was the case, as pumas were no more responsive to small dog than to frog playbacks on any behavioral measure (see below).

**Figure 2:**
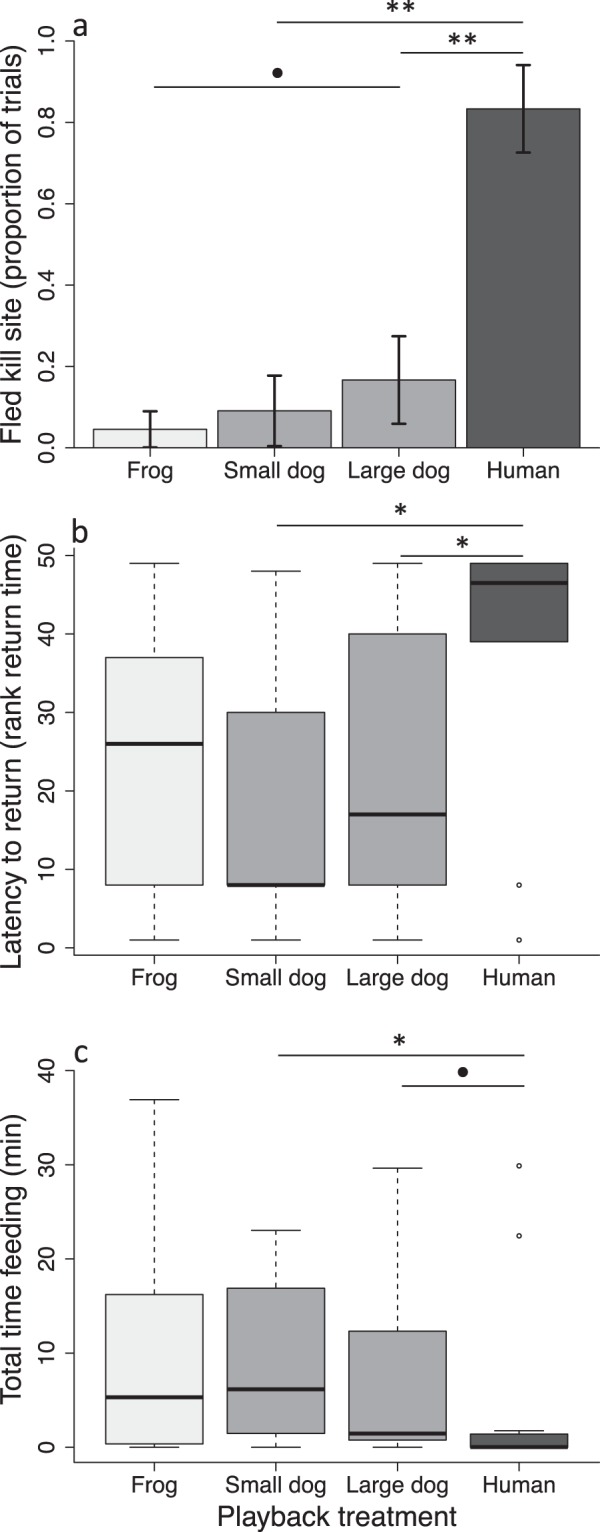
Puma responsiveness to domestic dog vocalizations. Responses to dogs are shown relative to known threatening (human) and non-threatening (frog) stimuli, as measured by (**a**) proportion (±1 proportional standard error^44^) of individual pumas that fled their kill site upon first hearing a playback, (**b**) latency to return (rank time) to the kill site after initial exposure, and (**c**) the total time spent feeding during the first 24 h of a given playback treatment. Data in (**b**,**c**) are presented as box plots, with the median indicated by the bold line within the box. Horizontal lines connecting treatments indicate significance level of difference between treatments: **p < 0.01; *p < 0.05; ^•^0.10 > *p* > 0.05.

Playback treatment had a significant effect on the likelihood of a puma fleeing its kill site (Fig. 2a; Binomial GLMM: Wald’s χ^2^ = 27.09, df = 3, *p* < 0.001; Supplementary Table S2). Pumas were significantly more likely to flee in response human playbacks (83% of trials) than to either small dogs (9%; Dunnett contrast: *z* = −3.26, *p* = 0.006) or large dogs (17%; *z* = −2.62, *p* = 0.003). Pumas were no more likely to flee in response small dogs than to frogs (5% of trials; *z* = 1.34, *p* = 0.253), and showed a weak and non-significant tendency to flee more often in response to large dogs than to frogs (*z* = 1.89, *p* = 0.089). We did not detect effects of building density, distance to road, or exposure on the likelihood of a puma fleeing its kill site (Supplementary Table S2).

While pumas abandoned their kill (i.e., did not return) in response to human playbacks in 42% of trials, abandonment only occurred in response to a single large dog trial (8%) and never in response to small dogs. Pumas abandoned their kill following frog playbacks in 14% of trials. For trials in which the puma did return to its kill, individuals typically took longer to do so following human playbacks (median = 20 min, range = 0–257) than following large dogs (median = 1 min, range = 0–60), small dogs (median = 1 min, range = 0–347), or frogs (median = 1 min, range = 1–40). The overall latency for pumas to return to their kill after initial exposure to a playback treatment was therefore significantly greater following humans than following large dogs (Mann-Whitney U-test; Bonferroni-adjusted *p* = 0.037) or small dogs (Bonferroni-adjusted *p* = 0.035), while pumas took no longer to return following large or small dogs than following frogs (all *p* ≥ 0.457; Fig. 2b).

Playback treatment affected the total amount of time that pumas spent feeding over a 24-h period (Fig. 2c; LMM: Wald’s χ^2^ = 8.02, df = 3, *p* = 0.046; Supplementary Table S3), with pumas spending significantly less time feeding when exposed to humans (mean ± SE = 4.6 ± 2.9 min) than when exposed to small dogs (9.2 ± 2.7 min; Dunnett contrast: *z* = 2.27, *p = *0.042). Pumas also tended to spend less time feeding when exposed to humans relative to large dogs (7.0 ± 2.7 min) though this difference was not significant at α = 0.05 (*z = *1.99, *p* = 0.084). We found no difference in total time spent feeding over a 24-h period when comparing exposure to frog playbacks (feeding time = 10.9 ± 2.7 min) with exposure to small dogs (*z = *0.027, *p* = 1.0) or large dogs (*z = *−0.516, *p* = 0.836). We did not detect effects of building density, distance to road, or exposure on the total amount of time pumas spent feeding (Supplementary Table S3).

## Discussion

We found little evidence that the perceived presence of dogs displaces pumas from their kills or significantly affects their feeding behavior while at kill sites. Dog playbacks had only minimal effects on puma behavior, with responses to small dogs being indistinguishable from controls on all behavioral measures. Pumas were slightly (though not significantly) more likely to flee in response to large dogs (17% of trials) than to frogs (5% of trials) but returned just as quickly to their kills and spent just as much time feeding. By contrast, responses to dogs (both large and small) were consistently lower than to humans, a known source of fear for pumas in our study population^26,36^. We have previously shown that pumas respond strongly to perceived risk from humans, leading to displacement from and reduced feeding time at kills, and likely driving cascading effects by forcing pumas to increase their kill rate on prey^12,26^. The present study indicates that pumas do not perceive dogs as major threats (no significant difference in responsiveness to frog and large dog cues) nor as reliable indicators of risk from humans (low responsiveness to small and large dog cues, despite their association with humans). The presence of dogs may therefore be unlikely to result in community level effects similar to those driven by fear of humans.

Given that domestic dogs are the most common carnivore in our study area^37^, it is unlikely that our results can be explained by pumas being unfamiliar with dog cues, and thus unable to associate them with threat. Rather, it is likely that the actual threat posed by dogs as competitors or predators is relatively low in our study area, and that puma responses to dogs represent this limited threat. Large populations of feral or free-ranging dogs are known to pose a substantial threat to some wildlife populations^1,7^, and correlational studies suggest that pumas may avoid areas where feral dogs are abundant^4,8^. However, in central California feral dogs are uncommon and the large majority of dogs in wildland areas are associated with humans^31^, leading to relatively high exposure to dog cues^37^, but likely limiting the potential for threatening interactions between dogs and wildlife^30^. Recreational puma hunting is also banned in California, and pumas in this area may therefore perceive less threat from dogs than elsewhere in western North America where recreational puma hunting with dogs is permitted. Further, some large carnivores may perceive dogs as prey^1,18^, reducing the likelihood of perceived threat. Though uncommon, pumas in our study population occasionally take wild canids (i.e., coyotes *Canis latrans* and gray foxes *Urocyon cinereoargenteus*^48^), and the perception of canids as potential prey could extend to domestic dogs.

Interestingly, we found no evidence that the strong association between dogs and humans in our study area results in pumas using dog cues as an indicator of human presence. If dogs served as a proxy for people, we would expect comparable puma responses to both dog and human playbacks, rather than the consistently greater response to humans observed here. Studies from the United Kingdom^39^, Uganda, South Africa, and the USA (LYZ and MC, unpublished data) have similarly found a lack of correspondence between wildlife responses to dog and human cues, indicating that, for many wildlife populations, dogs may serve as a poor indicator of human presence. Most dog breeds are highly vocal, and where dogs are common, pumas and other wildlife may learn to discriminate dog cues from human presence, or simply become habituated to frequent dog barks.

While pumas rely on multiple sensory modalities (acoustic, visual, and olfactory) to assess risk, our experiment focused specifically on acoustic cues, which provide perhaps the most straightforward method of simulating the immediate presence of a predator or competitor^39^. As with all mammals, pumas also use sight and scent to communicate with conspecifics^49^ and to eavesdrop on potential predators and competitors^50,51^. Simulating the visual presence of a predator has been successful in evaluating prey responses to predators^52^, but is difficult to effectively implement in the field, particularly over large spatial scales. Predator scent cue experiments are less difficult to implement, but because the decay of volatile scent molecules can cause rapid changes in the information content of the scent source^53,54^, such experiments are often difficult to interpret^51,55^. Correspondingly, the very few experimental studies examining the effect of human scent on wildlife behavior are similarly ambiguous^56–58^. By contrast, hearing a predator leaves little doubt that the predator is currently present, and dozens of studies (reviewed in^41^) have accordingly demonstrated that mammalian wildlife, including pumas^26,38^, respond fearfully to the vocalizations of threatening species. Thus, limited responsiveness by pumas to domestic dogs provides strong evidence that pumas in our study population do not perceive dogs as a substantial threat. How wildlife interpret and respond to combinations of multiple cue types from threatening species will be an interesting topic for future research, but was beyond the scope of the present study.

As noted in the Methods section, all kill sites were necessarily visited by a human to set up the experimental system, which means that some cues of human presence, including scent, may have been detectable at all sites regardless of the playback treatment presented. The degree to which responses to playback treatments were affected by any residual human cues at playback sites is unknown. Scent in particular is unlikely to have had a strong effect on puma responses. A recent experiment^59^ demonstrates that pumas are less likely to detect scent marks from other pumas, even at close range, unless they are also paired with a visual cue (a “scrape”). However, if we conservatively assume that human cues were detectable at kill sites, then dog vocalizations may have been perceived as being associated with humans, which is indeed the only relevant context in our study system, where feral dogs are largely absent. This is also the most relevant context to determining whether restricting (human-associated) dogs from protected areas will benefit pumas^2,28,31^.

Our findings contribute to a growing body of evidence that humans have a substantially greater impact on wildlife behavior than do other large carnivore predators/competitors^32,39^. In the only other study to experimentally compare the responses of a mammalian carnivore to both humans and large carnivores, Clinchy *et al*.^39^ showed that antipredator responses to humans by European badgers (*Meles meles*) far exceed responses to a suite of large carnivore predators, including domestic dogs. Large carnivores and mesocarnivores experience rates of human-caused mortality that far exceed those from non-human predators/competitors^23^, and these findings are consistent with carnivores perceiving humans as the most lethal threat. Using camera traps in protected areas across eastern North America (where free-ranging dogs are also rare), Parsons *et al*.^30^ found that several wildlife species (white-tailed deer *Odocoileus virginianus*, eastern gray squirrels *Sciurus carolinensis*, and raccoons *Procyon lotor*), all of which are hunted by humans, similarly showed significantly stronger antipredator responses to humans (with or without dogs) than to dogs alone, indicating that greater perceived threat from humans than from dogs is generalizable across wildlife species.

With an estimated global population of over 700 million domestic dogs^7^, the impact of dogs on wildlife in protected areas has become a topic of considerable management interest^28,29^. Several recent studies suggest that recreationalists with dogs have greater impacts on wildlife than do people without dogs^2,28,30,31,34,60^, and in many cases, management policies restricting dogs in protected areas are based on this perception of additional impacts from dog presence^29,32^. Our results indicate that, for pumas in North America, where feral or free-ranging dogs are relatively rare, the presence of dogs (particularly on leash) may have limited additional impacts beyond those caused by the presence of humans, and thus that restricting dogs while allowing human recreation may not substantially benefit pumas. These findings may not apply to other wildlife species, e.g., small mammals for which domestic dogs pose a direct predatory threat^2^ (but see^30^). However, where the primary management goal is to protect large carnivore populations in or near human-dominated landscapes, limiting human activity overall may be the most effective way to minimize disturbance to large carnivores.

## Supplementary information

Supplementary Information

Supplementary Data File 1

## Data Availability

All data necessary to conduct the analyses presented here are provided in the Supplementary Material (Supplementary Data File 1).
