# Supplementary material for: Humans, but not their dogs, displace pumas from their kills: An experimental approach
Source: Sci Rep. 2019 Aug 21;9:12214. doi: 10.1038/s41598-019-48742-9 (PMC6704098; doi:10.1038/s41598-019-48742-9)
Supplement: Supplementary file 1 — Supplementary Information [file 41598_2019_48742_MOESM1_ESM.docx]

*Supplementary Information for*

**Humans, but not their dogs, displace pumas from their kills: An experimental approach**

Justin P. Suraci^1*^, Justine A. Smith^2^, Michael Clinchy^3^, Liana Y. Zanette^3^, Christopher C. Wilmers^1^

^1^Center for Integrated Spatial Research, Environmental Studies Department, University of California, Santa Cruz, CA 95064, USA

^2^Department of Environmental Science, Policy, and Management, University of California, Berkeley, CA 94720, USA

^3^Department of Biology, Western University, London, ON N6A 5B7, Canada

* Corresponding author. Email: [justin.suraci@gmail.com](mailto:justin.suraci@gmail.com); Phone: 831-999-0492

*This document includes*:

Supplementary Tables S1-S3

**Table S1.** Summary (**a**) of spatial covariates measured at each puma kill site, along with results of linear mixed effects models and post-hoc tests comparing (**b**) distance to major road and (**c**) building density (number of buildings within 500 m) between playback treatments.

| **(a) Data summary** |  |  |  |  |  |  |  |  |  |  |  |
| --- | --- | --- | --- | --- | --- | --- | --- | --- | --- | --- | --- |
|  |  |  | *Distance to major road (m)* | | | |  | *Building density* | | | |
| **Playback treatment** | **n** |  | **Mean** | **SD** | **Min** | **Max** |  | **Mean** | **SD** | **Min** | **Max** |
| Frog | 22 |  | 1201.0 | 854.1 | 60.4 | 3390.5 |  | 42.5 | 97.0 | 0.0 | 294.0 |
| Human | 12 |  | 1201.7 | 630.2 | 350.9 | 2664.2 |  | 39.8 | 130.5 | 0.0 | 454.0 |
| Large Dog | 12 |  | 958.2 | 1078.7 | 60.4 | 3390.5 |  | 50.9 | 104.2 | 0.0 | 284.0 |
| Small Dog | 11 |  | 690.0 | 999.4 | 60.4 | 3390.5 |  | 35.4 | 78.3 | 0.0 | 261.0 |

| **(b) Distance to road** |  |  |  |  |
| --- | --- | --- | --- | --- |
| *Overall model (n = 57)* |  |  |  |  |
|  | **Wald's χ2** | **DF** | **p-value** |  |
| Playback treatment | 7.782 | 3 | 0.051 |  |
|  |  |  |  |  |
| *Tukey's post-hoc tests* |  |  |  |  |
|  | **Difference** | **SE** | **z-value** | **p-value** |
| Human-Frog | -0.068 | 0.201 | -0.341 | 0.986 |
| Large Dog-Frog | 0.303 | 0.224 | 1.353 | 0.523 |
| Small Dog - Frog | 0.562 | 0.216 | 2.602 | *0.045* |
| Large Dog-Human | 0.371 | 0.267 | 1.389 | 0.500 |
| Small Dog-Human | 0.631 | 0.258 | 2.442 | *0.068* |
| Small Dog-Large Dog | 0.259 | 0.225 | 1.153 | 0.651 |
|  |  |  |  |  |
|  |  |  |  |  |
| **(c) Building density** |  |  |  |  |
| *Overall model (n = 57)* |  |  |  |  |
|  | **Wald's χ2** | **DF** | **p-value** |  |
| Playback treatment | 15.571 | 3 | 0.001 |  |
|  |  |  |  |  |
| *Tukey's post-hoc tests* |  |  |  |  |
|  | **Difference** | **SE** | **z-value** | **p-value** |
| Human-Frog | 0.046 | 0.112 | 0.409 | 0.976 |
| Large Dog-Frog | -0.252 | 0.125 | -2.014 | 0.179 |
| Small Dog - Frog | -0.447 | 0.121 | -3.696 | *0.001* |
| Large Dog-Human | -0.298 | 0.150 | -1.995 | 0.186 |
| Small Dog-Human | -0.493 | 0.145 | -3.410 | *0.003* |
| Small Dog-Large Dog | -0.195 | 0.126 | -1.546 | 0.404 |

**Table S2.** Results of the generalized linear mixed effects models testing the effects of playback treatment on the likelihood of a puma fleeing its kill site. Dunnett contrasts are presented for specific playback treatment pairs of interest.

| *Full model (n = 57)* |  |  |  |  |
| --- | --- | --- | --- | --- |
|  | **Wald's χ2** | **DF** | **p-value** |  |
| Treatment | 27.090 | 3 | <0.001 |  |
| Exposure | 0.032 | 1 | 0.857 |  |
| Distance to road | 0.018 | 1 | 0.893 |  |
| Building density | 0.037 | 1 | 0.848 |  |
|  |  |  |  |  |
| *Dunnett Contrasts* |  |  |  |  |
|  | **Difference** | **SE** | **z-value** | **p-value** |
| *Comparing human (n = 12) vs. small (n = 11) and large (n = 12) dogs* | | | | |
| Small Dog - Human | -40.090 | 12.31 | -3.255 | 0.002 |
| Large Dog - Human | -26.260 | 10.01 | -2.623 | 0.015 |
|  |  |  |  |  |
| *Comparing frog (n = 22) vs. small (n = 11) and large (n = 12) dogs* | | | | |
| Small Dog - Frog | 15.970 | 11.91 | 1.342 | 0.253 |
| Large Dog - Frog | 31.950 | 16.93 | 1.886 | 0.089 |

**Table S3.** Results of the linear mixed effects models testing the effects of playback treatment on the total time pumas spent feeding over a 24-hour period. Dunnett contrasts are presented for specific playback treatment pairs of interest.

| *Full model (n = 57)* |  |  |  |  |
| --- | --- | --- | --- | --- |
|  | **Wald's χ2** | **DF** | **p-value** |  |
| Treatment | 8.023 | 3 | 0.046 |  |
| Exposure | 1.787 | 1 | 0.181 |  |
| Distance to road | 1.000 | 1 | 0.317 |  |
| Building density | 0.722 | 1 | 0.396 |  |
|  |  |  |  |  |
| *Dunnett Contrasts* |  |  |  |  |
|  | **Difference** | **SE** | **z-value** | **p-value** |
| *Comparing human (n = 12) vs. small (n = 11) and large (n = 12) dogs* | | | | |
| Small Dog - Human | 3.685 | 1.62 | 2.279 | 0.042 |
| Large Dog - Human | 3.164 | 1.59 | 1.992 | 0.084 |
|  |  |  |  |  |
| *Comparing frog (n = 22) vs. small (n = 11) and large (n = 12) dogs* | | | | |
| Small Dog - Frog | 0.069 | 2.58 | 0.027 | 1.000 |
| Large Dog - Frog | -1.295 | 2.51 | -0.516 | 0.836 |
